# Through-the-cholangioscope metal biliary stent placement as a novel endoscopic technique for bile duct strictures

**DOI:** 10.1055/a-2241-1916

**Published:** 2024-02-15

**Authors:** Carlos Robles-Medranda, Maria Egas-Izquierdo, Juan Alcívar-Vásquez, Miguel Puga-Tejada, Martha Arevalo-Mora, Domenica Cunto, Jorge Baquerizo-Burgos

**Affiliations:** 1 Gastroenterology, Instituto Ecuatoriano de Enfermedades Digestivas – IECED, Guayaquil, Ecuador

The management of biliary strictures remains challenging. Biliary drainage via endoscopic retrograde cholangiopancreatography (ERCP) and endoscopic ultrasound (EUS) are the currently preferred approaches 1 2 3 , but advances in cholangioscopy are allowing new tools to be considered 4 . We present our experience using a novel through-the-cholangioscope self-expandable metal stent (TTC-SEMS; Micro-Tech, Nanjing, China) ( Video 1 ).

Placement of through-the-cholangioscope metal stents in two patients with biliary strictures.Video 1

A 61-year-old woman who had had a plastic stent in place for 3 months for a long stricture in the common bile duct presented with weight loss and right upper quadrant abdominal pain. EUS revealed biliary tract dilatation and a Bismuth-Corlette type 1 stricture ( Fig. 1 ). The patient refused to undergo surgery and instead underwent artificial intelligence (AI)-assisted cholangioscopy (AIworks-Cholangioscopy; mdconsgroup, Ecuador). A malignancy was detected, and a tissue sample was obtained, with rapid on-site evaluation being positive for malignancy. A 5 Fr, 10-mm × 6-cm TTC-SEMS was delivered, without technical difficulties, with its position confirmed by direct visualization ( Fig. 2 ). The patient’s symptoms resolved within 24 hours of the procedure.

**Fig. 1:**
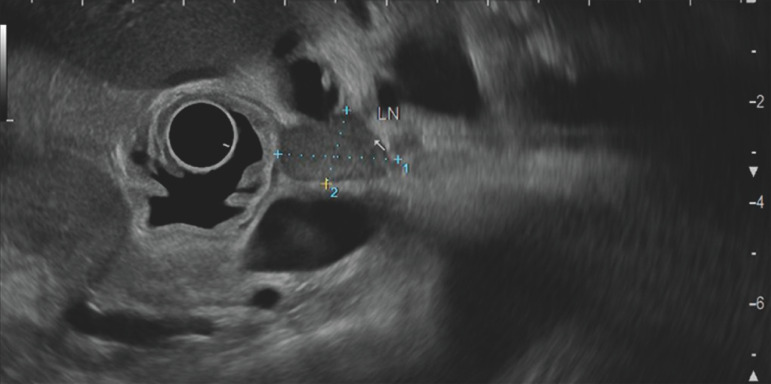
Endoscopic ultrasound image showing dilatation of the biliary tract with hypoechogenic irregular thickening of the distal common bile duct.

**Fig. 2:**
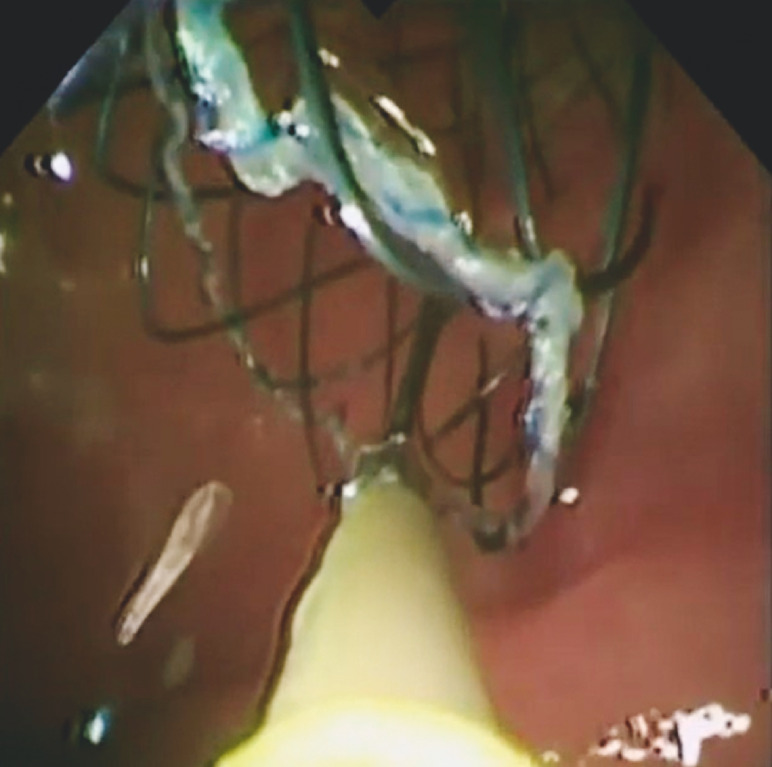
Cholangioscopic image showing placement of a through-the-cholangioscope metal stent in a patient with cholangiocarcinoma.

A 74-year-old man presented with a 2-month history of jaundice, weight loss, and ascites. ERCP revealed a type I Bismuth-Corlette stricture ( Fig. 3 ), and a plastic stent was placed. EUS revealed a 20 × 24-mm hypoechoic irregular lesion. Biliary drainage was performed via a gastric approach with a 10 × 10-mm lumen-apposing metal stent (LAMS), and the plastic stent was removed 1 month later. AI-assisted cholangioscopy detected a digitiform lesion ( Fig. 4 and Fig. 5 ); biopsy confirmed cholangiocarcinoma. Radiofrequency ablation was performed, with subsequent placement of a 5 F, 10-mm × 6-cm TTC-SEMS. No adverse events were reported within 48 hours.

**Fig. 3:**
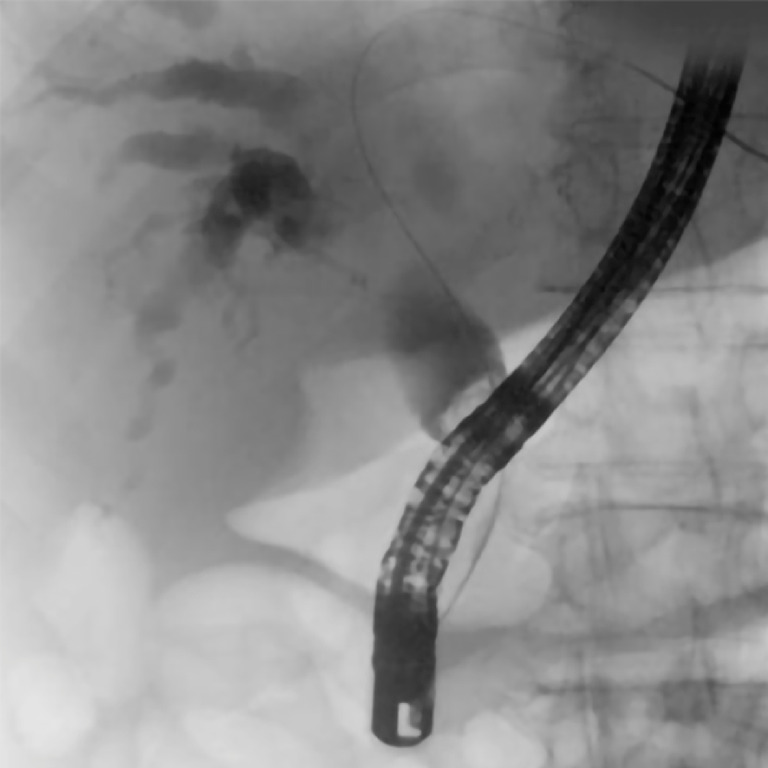
Fluoroscopic image showing a type I Bismuth-Corlette stricture.

**Fig. 4:**
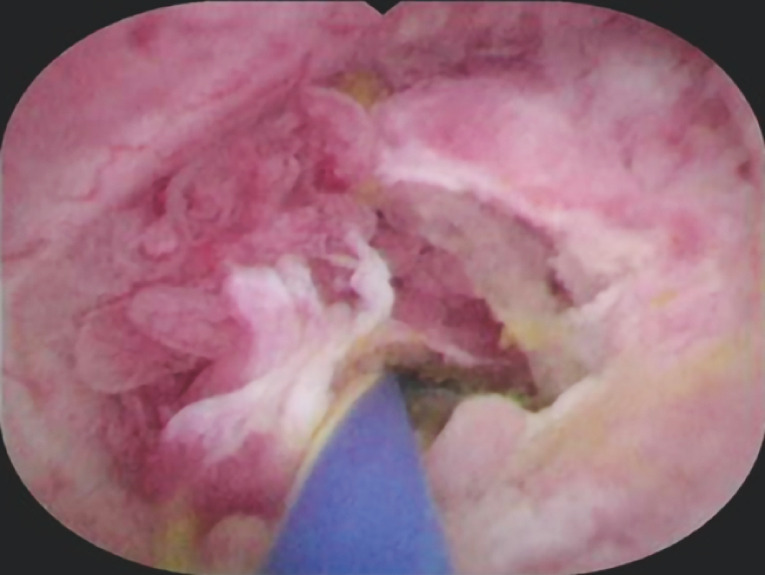
Cholangioscopic image of a digitiform mucosal lesion observed during the cholangioscopic procedure.

**Fig. 5:**
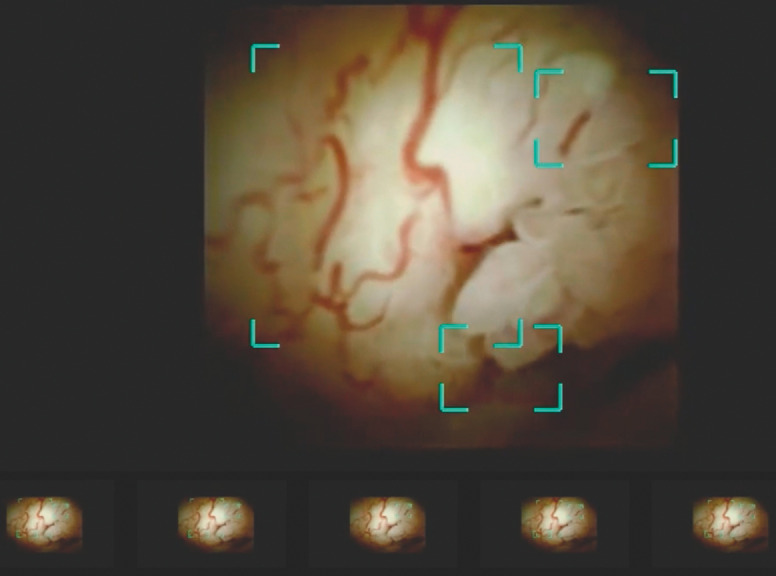
Artificial intelligence (AI)-assisted detection of a lesion suggestive of neoplasia, using a novel artificial intelligence model, during a cholangioscopic procedure.

We achieved both technical (correct stent positioning under direct visualization) and clinical (symptom reduction) success. The use of AI significantly contributed to effective tissue sampling. No adverse events were reported. One advantage of TTC-SEMSs is their placement under direct visualization, resulting in a reduction in fluoroscopic radiation – a first step for the future of stent placement procedures without fluoroscopic guidance. The use of TTC-SEMSs is promising and needs to be explored for the management of biliary strictures.

Endoscopy_UCTN_Code_TTT_1AR_2AZ

## Notes

Endoscopy E-Videos https://eref.thieme.de/e-videos
*E-Videos* is an open access online section of the journal *Endoscopy* , reporting on interesting cases and new techniques in gastroenterological endoscopy. All papers include a high-quality video and are published with a Creative Commons CC-BY license. Endoscopy E-Videos qualify for HINARI discounts and waivers and eligibility is automatically checked during the submission process. We grant 100% waivers to articles whose corresponding authors are based in Group A countries and 50% waivers to those who are based in Group B countries as classified by Research4Life (see: https://www.research4life.org/access/eligibility/ ). This section has its own submission website at https://mc.manuscriptcentral.com/e-videos .
